# Recommendations for resistance training in patients with fibromyalgia

**DOI:** 10.1186/s13075-015-0782-3

**Published:** 2015-09-17

**Authors:** Kim Dupree Jones

**Affiliations:** Oregon Health & Science University, School of Nursing, Mail Code SN-ORD, 3455 SW US Veterans Hospital Road, Portland, OR 97239 USA

## Abstract

It may seem counter-intuitive to purposely stress muscle in patients who have muscle pain. However, a growing body of evidence challenges the assumption that resistance (strength) training worsens muscle pain in people with fibromyalgia (FM). In fact, the latest evidence indicates that when resistance training is tailored to individual needs, people with FM can obtain worthwhile improvements in FM severity. Clinicians need a deeper understanding of how resistance training helps people with FM, so as to prescribe more specific, personalized resistance training to their patients.

Resistance training is a type of exercise in which progressive resistance is used to improve muscle strength, endurance, power, or a combination. Resistance can be manipulated with free or machine weights, bands, or elastic tubing, or even with one’s own body weight. Resistance training not only builds muscle strength and mass, it also produces improvements in balance, coordination, and agility.

In an article published recently in *Arthritis Research & Therapy*, Larsson et al. [1] report the results of a multicenter, parallel randomized controlled trial (RCT) in which they compared group-based progressive resistance (strength) training versus relaxation training in 130 Swedish women with fibromyalgia (FM). The study was based on a “person-centered” model of exercise. This model actively involves the patient in planning the treatment, and optimizes self-confidence for exercise. Small groups of five to seven women exercised together under the supervision of a physiotherapist at a local gymnasium, twice weekly for 15 weeks. Starting loads were low (40 % one repetition maximum (1RM), the heaviest weight a person can lift or move in one contraction) and slowly progressed to 80 % 1RM. However, patients could decline increasing loads if they were unsure they could manage the new load. Immediately post intervention, the resistance training group—compared with the relaxation training group—demonstrated significant improvements in isometric knee and elbow extension, 6-minute walk, and health status (Fibromyalgia Impact Questionnaire). At 13–18 months post intervention, differences were no longer found between the groups on any measure, underscoring the difficulty in adopting exercise outside of a formal program.

Additional support for the benefits of resistance training in FM can be found in a recent Cochrane Database review [2]. The authors concluded that resistance training improves multidimensional function, pain, tenderness, and muscle strength in women with FM. However, the level of evidence remains “low” owing to the small number of resistance training RCTs to date [3–7]. Moreover, men have yet to be studied and minority representation is small. The optimal training frequency, intensity, timing, and progression are not yet fully understood in FM. This gap is due, in part, to the heterogeneity of fitness levels and symptom burden in people with FM. There is good evidence for individualizing the prescription of exercise with the philosophy of “starting low and going slow” during progression. Realistically, most patients will have difficulty transferring these results into a long-term habit outside the support of a formal study. Beyond the results reported here, there is evidence for the benefits of resistance training in healthy individuals in terms of body composition, muscle strength, age-related muscle loss, and all-cause mortality [8, 9].

Having FM poses hurdles that need to be to overcome before reaping the rewards of resistance training. People with FM are less physically active compared with age-matched controls [10]. Deconditioned muscle is a potent pain generator owing to delayed-onset muscle soreness (DOMS). This is a result of an inflammatory response during the repair and adaption process of building muscle (e.g., microtrauma, repair, and growth) [11, 12]. Not surprisingly, sedentary persons may have difficulty in initiating or maintaining an exercise program because of both immediate pain and DOMS. Simply put, being inactive will eventually lead to more pain on exertion, and for many will result in a symptom flare.

Patients rarely have access to an academic RCT like that of Larsson et al. Clinicians therefore need to be able to give patients safe, evidence-based exercise advice. Here is some pragmatic guidance: do not expect resistance training to be a replacement for medications; instead think of exercise as a key part of the overall interdisciplinary management plan.

The following presents some general advice for patients. Have a well-defined training goal in mind and focus on training consistently rather than intensively. Minimize eccentric muscle loading (a major cause of DOMS) by limiting overhead arm work and exercises done with limbs farther away from the body’s midline. Do not attempt to do strength training during a symptom flare. Do not attempt high-intensity, power-based workouts (e.g., pylometrics, CrossFit, bootcamps). Limit pain-provoking postures by working within the natural joint lines; this reduces the risk of aggravating tendinopathies or overextending hypermobile joints. Avoid the urge to overtrain on days you feel better. Be proactive by providing FM-specific exercise advice to a fitness trainer [13]. Work out at home with DVDs especially formulated for FM patients [14]. Lastly, link an exercise with an activity you like or do regularly to increase the likelihood that exercise will become a life-long habit.

## Abbreviations

1RM: One repetition maximum
DOMS: Delayed-onset muscle soreness
FM: Fibromyalgia
RCT: Randomized controlled trial
